# Resource Availability and Competition Shape the Evolution of Survival and Growth Ability in a Bacterial Community

**DOI:** 10.1371/journal.pone.0076471

**Published:** 2013-09-30

**Authors:** Minna Pekkonen, Tarmo Ketola, Jouni T. Laakso

**Affiliations:** 1 Integrative Ecology Unit, Centre of Excellence in Biological Interactions, Department of Biosciences, University of Helsinki, Helsinki, Finland; 2 Centre of Excellence in Biological Interactions, Department of Biological and Environmental Science, University of Jyväskylä, Jyväskylä, Finland; Leiden University, Netherlands

## Abstract

Resource availability is one of the main factors determining the ecological dynamics of populations or species. Fluctuations in resource availability can increase or decrease the intensity of resource competition. Resource availability and competition can also cause evolutionary changes in life-history traits. We studied how community structure and resource fluctuations affect the evolution of fitness related traits using a two-species bacterial model system. Replicated populations of *Serratia marcescens* (copiotroph) and *Novosophingobium capsulatum* (oligotroph) were reared alone or together in environments with intergenerational, pulsed resource renewal. The comparison of ancestral and evolved bacterial clones with 1 or 13 weeks history in pulsed resource environment revealed species-specific changes in life-history traits. Co-evolution with *S. marcescens* caused *N. capsulatum* clones to grow faster. The evolved *S. marcescens* clones had higher survival and slower growth rate then their ancestor. The survival increased in all treatments after one week, and thereafter continued to increase only in the *S. marcescens* monocultures that experienced large resource pulses. Though adaptive radiation is often reported in evolution studies with bacteria, clonal variation increased only in *N. capsulatum* growth rate. Our results suggest that *S. marcescens* adapted to the resource renewal cycle whereas *N. capsulatum* was more affected by the interspecific competition. Our results exemplify species-specific evolutionary response to both competition and environmental variation.

## Introduction

Both biotic and abiotic environment shape the evolution within populations and communities. Biotic environmental factors include interactions with other organisms, such as intra- and interspecific competition, predation, parasitism, or demographic changes within population [1,2]. Abiotic environment contains physical and climatic factors, and also depending on definition, non-renewable resources [1,2]. Often abiotic and biotic environmental factors are interconnected: abiotic factors affect interactions between organisms, and the activity of organisms changes abiotic environmental conditions [1,3]. Moreover, abiotic and biotic environmental conditions vary in time and/or space [2,4]. To understand how environment shapes evolution within communities there is a need for experiments manipulating both community structure and abiotic conditions. This is utmost important as the eco-evolutionary processes caused by environmental factors cannot always be separated from the selection pressures intrinsic to communities. For example, species interactions could impede or facilitate the evolution on abiotic conditions when species exist together [5–7]. Indeed, the very scarce experimental evidence point to the direction that abiotic selection pressures can be diminished or strengthened in the presence of competitor species [7,8].

One profound abiotic condition (especially in bacteria and plants) is the quantity of nonliving resources. Resource availability can fluctuate so that periods of resource abundance are followed by periods of resource scarcity. Also the magnitude of fluctuations can vary: in some environments fluctuations are subtle, whereas in others resource availability alternates between gluttony and famine. In general different traits could be beneficial depending on the resource availability. In environments where resource fluctuations are frequent and severe, the high responsiveness for suddenly released resources is expected to be under strong selection pressure [9]. Such environments could select for high growth rate during resource abundance. A well-known example of an environment where resources become available seasonally and in large quantities causing pronounced changes in local communities are algal blooms in eutrophic water bodies [10]. In some environments survival through prolonged adverse conditions is crucial, such as arid regions characterized by prolonged periods of drought with occasional rains causing sudden resource increase [11]. When resources become available in rare pulses selection can favour the ability to efficiently utilize the resource inflow, but also traits that enable growth and survival in dense population or when resources are scarce can be important [9,11,12]. However, a trade-off between maximizing growth rate and yield or survival may hinder investing in these traits simultaneously [13–15].

In addition to the environment driven evolution also interactions between individuals can cause evolutionary changes in life-history traits. The intensity and direction of interactions are often tightly linked to resource availability. Already Darwin realized how important “the struggle for life”, essentially resource competition, is in shaping the evolution of all organisms when resources are limited (Ch. 3 in [16]). The general prediction based on classical competitive exclusion principle is that if species share the same limiting resource they cannot coexist indefinitely, but competitively superior will outcompete the weaker ones [17,18]. In a long enough time the selection pressures caused by competition can cause evolutionary changes in resource preferences or growth strategies of organisms [19]. On the other hand, fluctuations in environmental conditions can also enable the coexistence of species that differ in their growth strategies [5,18,20,21]. Coexistence is possible if e.g. other species grows fast on abundant resources, whereas the other is able to grow on scarce resources and the resource availability varies [5,18,20]. However, evolutionary changes that cause niche expansion and result in a significant overlap of growth strategies can make the coexistence unlikely [4].

How the co-evolution affects traits is crucially dependent on the type of species interactions. When two species compete for the same limiting resource the reduction of the niche overlap may lead to evolutionary changes in both species. Alternatively the competitively dominant species does not change and is maybe even able to expand its niche, and thus reduces niche space available for the other species. This causes selection pressure to the inferior competitor to adapt to utilize whatever resources are left available. Resource competition can thus lead to niche expansion or shift in niche preferences [4]. In a situation where resources are abundant and the resource competition is weak, the niche expansion might not be selectively advantageous even though free niches were available [22]. Especially, in the absence of species interactions resource abundance could favour the evolution of specialists capable of utilizing only a narrow range of available resources, evolving at only the most productive environments, not extremes [23]. In this scenario a large proportion of potential niche space is left unused. However, even in monocultures temporal resource scarcity or overcrowding can both potentially increase density dependent intraspecific competition [1]. In a long enough time, competition between conspecifics can push forward evolutionary changes that lead to niche expansion and the utilization of all potential niches [1,24]. This type of adaptive radiation is extensively studied using microbes as model organisms (reviewed in [25]).

To explore the effects of resource fluctuations and intra- vs. interspecific interactions on evolutionary changes in life-history traits (growth rate, yield, survival and biofilm production) we exposed bacterial species living either in monocultures or two-species communities to inter-generational, low frequency resource pulses. The replicated (n=3) experiment lasted 13 weeks enabling the occurrence of evolutionary changes in life history traits. The two bacterial species were chosen on a basis that they differ in their growth strategies, but are able to exploit same resources [26,27]. *Serratia marcescens* grows quickly when resources are abundant, but on low resource levels growth rate is slow. *Novosphingobium capsulatum* has slower growth rate than *S. marcescens* when resource are abundant, but it grows well even when resources are scarce. The earlier work on these species shows that at population level the evolutionary history in pulsed resource environment with relatively long interpulse periods increased the mean survival of both species without a distinct trade-off with growth rate [28]. In the present work we ask two questions that were not addressed before on these same bacterial strains: 1) Can the clonal variation in population samples mask evolutionary changes in traits? 2) How does the average performance of evolved bacterial clones from two-species communities and from monocultures differ, when the competitor is absent?

Our general hypothesis on the effects of resource fluctuations on life-history trait evolution is that in large resource pulse environments fast growth during resource abundance is selected for, whereas survival during resource scarcity is under selection in environment with smaller resource pulses. Our earlier work shows that on population level the general response to rare resource fluctuations in these species was increase in survival with no clear cost in growth performance [28]. Both *S. marcescens* and *N. capsulatum* grew to larger population size in monocultures than when grown together [28], which indicates that these species compete with each other [29,30]. Competition between or within species can either speed up or hinder adaptation to changing environmental conditions ( [31] and references therein). Competition may reduce population abundance and increase the risk of extinction when environmental conditions change, but competition can also expedite adaptation if competition and environmental change cause selective pressure to parallel direction [31]. By comparing the performance of bacterial clones with evolutionary history in either monocultures or two-species communities, we can disentangle the possible effect of interspecific competition on the evolution of measured life-history traits. We hypothesize that interspecific competition potentially affects traits where these species have niche overlap.

In diverse multi-clone populations few clones can potentially dictate most of the observed growth patterns, which hinders the detection of evolutionary differences between treatments. Moreover, this is especially problematic in studies attempting to reveal trade-offs between traits. When the measurements are conducted at the population level clonal variation could allow some clones in the population to grow fast and others to contribute to high yield resulting in the apparent lack of a trade-off. It is possible, that trade-offs between life-history traits are manifested when the analysis level is changed from populations to clones. Because bacteria reproduce asexually the measured performance of bacterial clones can be used as an approximation of genetic variation within populations. Furthermore, monitoring the performance of several bacterial clones at different times of their evolutionary history allows testing if variation in measured life-history traits changes in response to selection. If the variation in some of the measured traits increases, it can indicate that several growth strategies are selectively advantageous simultaneously (disruptive selection), or alternatively selection is weak and variation increases due to mutation accumulation. Decrease in variation can denote directional or balancing selection. Directional selection can also lead to a situation where variation remains constant and only the trait mean changes.

## Methods

Study species *Serratia marcescens* (from American Type Culture Collection strain ATCC 13880) and *Novosphingobium capsulatum* (ATCC 14666) are heterotrophic, gram-negative, rod shaped bacteria that do not form spores. *Serratia marcescens* is facultative anaerobe and belongs to the family of Enterobacteriaceae [32–34]. The ATCC strain of *S. marcescens* was originally isolated from pond water. *Novosphingobium capsulatum* is aerobe and belongs to the family of Sphingomonadaceae [35,36]. The *N. capsulatum* strain was originally isolated from distilled water [35]. Species can be separated based on colony morphology: *S. marcescens* forms white, pink or red colonies whereas *N. capsulatum* forms yellow colonies when grown on Nutrient Broth agar plates. They have different growth responses to fresh cereal leaf medium: *N. capsulatum* grows faster than *S. marcescens* on low concentration, and *S. marcescens* grows faster on intermediate and high concentrations (mean ± SE Monod parameters estimated from measured growth rates in 0.1-1.0 gL^-1^ hay extract are: maximum growth rate r_max_ = 0.103 ± 0.047, half saturation constant K_s_ = 0.29 ± 0.36, and r_max_ = 0.418 ± 0.157, K_s_ = 1.72 ± 0.89 for *N. capsulatum* and *S. marcescens*, respectively [26]).

### Long-term experiment

*Serratia marcescens* and *N. capsulatum* were grown as monocultures and together in two-species communities in aquatic microcosms. The microcosms were filter-capped 250 ml cell culture bottles (Corning) containing 150 ml of phosphate buffered cereal leaf extract. The medium was prepared as follows: 1 gl^-1^ of cereal leaf powder (Ward’s natural science, Rochester, NY) was boiled for 10 min in deionised H_2_O (dH_2_O), cooled down, and filtered through a glass microfibre filter (GF/C, Whatman). The filtering procedure leaves 2.15 mgl^-1^ dry weight of cereal leaf powder to the final medium. Phosphate buffer adjusted to pH 7.5 (1.57 g of K_2_HPO_4_·3 H_2_O, 0.4 g of KH_2_PO_4_, 0.5 g of (NH_4_)_2_SO_4_, 0.1 g of MgSO_4_·7 H_2_O, 0.01 g of NaCl, and 0.023 g of CaCl_2_·2 H_2_O per 1 l of dH_2_O) was added to the medium. The medium was autoclaved at 121 °C for 20 min.

Bacteria were exposed to resource fluctuations once a week. Two distinct pulse magnitudes were used: in the resource renewal either 99.9% (large pulse) or 70% (small pulse) of the total volume of microcosms was replaced with fresh growth medium. For the starting populations, the bacteria were first cultivated for 3 days on agar plates (10 g of nutrient broth (Difco™, BD), 2.5 g of yeast extract (Scharlau Chemie S.A.), and 15 g of agar (Scharlau Chemie S.A.) in 1 l of dH _2_0). Then approximately 50 colonies were streaked from an agar plate and mixed in sterile phosphate buffered dH_2_O. The optical density (OD) of the bacteria water mixture was measured using wavelength 595 nm with Optiscan spectrophotometer and diluted to a final OD of 0.6, which equals 5.6×10^6^ ± 1.3×10^6,^ and 3.4×10^6^ ± 4.4×10^5^ CFUml^-1^ ± SE of *S. marcescens*, and of *N. capsulatum*, respectively (CFU = colony forming units, SE = standard error of mean). In the large resource pulse treatment, the starting population was 0.1% of the estimated maximum yield in hay extract medium (equalling 210 µl of the diluted bacteria water mixture). In the small resource pulse treatment the starting population size was 30% of the estimated maximum yield (equalling 6.3 ml of the diluted bacteria water mixture). For the two-species communities, the species were mixed in 1:1 biomass ratio. The total volume of the inoculate population was the same within each renewal regime.

The experiment was continued for 13 weeks. In the large resource pulse treatment the transfer volume was 150 µl, equalling a population size of approximately 2×10^5^ ± 2×10^4^ CFUml^-1^ ± SE in the *S. marcescens* monoculture, 6×10^4^ ± 8×10^3^ CFUml^-1^ ± SE in the *N. capsulatum* monoculture, and 1×10^5^ ± 3×10^4^ CFUml^-1^ ± SE in the two-species community. In the smaller resource pulse treatment the transfer volume was 45 ml, equalling approximately population size of 6×10^7^ ± 6×10^6^ CFUml^-1^ ± SE in the *S. marcescens* monoculture, 3×10^7^ ± 4×10^6^ CFUml^-1^ ± SE in the *N. capsulatum* monoculture, and 4×10^7^ ± 4×10^6^ CFUml^-1^ ± SE in the two-species community. All treatments had 3 replicates.

At each resource renewal a 0.5 ml sample of living cells from each microcosm was aseptically mixed in 0.6 ml of sterile freezing solution and stored in suspended animation at -70 °C. The freezing solution contained nutrient medium [10 g of nutrient broth (Difco™, BD), 1.25 g of yeast extract (Scharlau Chemie S.A.) in 1 l of dH _2_0], and glycerol (bidistilled 99.5% W/V, WVR) in volume ratio 1:5. During the long-term experiment the microcosms were kept at 25 °C. The relatively low concentration of the detritus resource and the volume to surface area ratio of the microcosms suggest that oxygen was available throughout the experiment in all parts of the microcosms.

### Fitness assays

The performance of 6–19 bacterial clones was measured for the ancestor strains of both species, and for the evolved strains from all selection lines after 1 week or 13 weeks in the long-term experiment. In the fitness assays bacterial clones were grown for one week on microtitre plates (Honeycomb 2, Thermo Electron Oy) in conditions comparable to experiment (growth medium, temperature), and ODs were recorded at 5 min intervals. The inoculate for the fitness assay was made as follows: the frozen bacteria samples from the long-term experiment were thawed and spread on agar plates (similar as in the long-term experiment) and incubated for 3 days at 25 °C. Each clone for the fitness assays was selected randomly, streaked from the plate, and mixed into 5 ml of phosphate buffered dH _2_0. The mixture was thoroughly shaken to ensure that bacterial biomass was evenly distributed to the liquid. 10 µl of this mixture was used as an inoculate population and mixed into 390 µl of the growth medium.

Maximum growth rate (h^-1^), yield, mortality, and the amount of biofilm produced during the measurement time were calculated based on OD data. An estimate of the maximum growth rate was the slope of the steepest regression line fitted to growth curve of 30 measurement points (equalling 2.5 h). Yield was maximum biomass. As mortality estimate we used biomass reduction after the maximum biomass was reached. Total mortality was calculated as maximum biomass minus end biomass, and proportional mortality was calculated as total mortality divided by the maximum biomass. Biofilm production was measured using crystal violet stain protocol ( [37], modified from [38]). After one week’s growth, 100 µl of 1% crystal violet solution (Tamro) was added to each well in the microtitre plate. After 10 min staining, all liquid was removed and the wells were rinsed three times with dH_2_O. Thereafter 450 µl of 96% ethanol was inserted to each well to dissolve the crystal violet stained bacteria from the walls. The amount of formed biofilm was estimated by measuring the absorbance of crystal violet ethanol solution at 460-580 nm for 20 h.

### Statistical analysis

For the statistical analysis we calculated the mean and variation in the performance of clones within population per each treatment and sampling time. We modeled the evolutionary changes during the long-term experiment using a General Linear Mixed Model (GLMM) procedure in SPSS v.16 (SPSS Inc., Chicago IL). We also modeled the effects of treatments on changes in clonal variation in measured traits using GLMM. Standard deviation (SD) of clones in measured traits was used as an estimate of clonal variation within populations. We constructed models separately for each species and trait (growth rate, yield, mortality, biofilm production). The model included a repeated factor and fixed factors with all 2- and 3-way interactions. The repeated factor was the time when clones were isolated (after 1 week or 13 weeks) and the experiment units (microcosms) were the repeated subjects. The time and the 2 treatments (resource pulse magnitude and species-composition) were set as the fixed effects. Unstructured covariance structure was used in all GLMM models. The comparison of ancestor to evolved bacterial clones could not be included in the GLMM, as there was not separate measurement for ancestor per each microcosm. Thus, the performance of ancestor and evolved clones with 1 week or 13 weeks evolutionary history in resource pulse environment were compared using Student’s two-tailed t-test with corrections for unequal sample sizes (formula 7.5, p. 191 in [39]). As a species-specific ancestor value we used the mean of 10 clone measurements. Treatment values for evolved clones were the estimated marginal means (EMM) and their standard errors (SE) from the GLMM (3-way interaction time x pulse x diversity). Due to multiple comparisons within each trait (4 treatments at each time-point), we used Bonferroni correction (0.05/4), resulting in the significance level for rejecting the null hypothesis as p < 0.0125. This correction reduces the likelihood of detecting false positive within four tests from 18.55% to 4,91%.

The genetic correlations between growth rate vs. yield, and growth rate vs. mortality were tested using a Pearson 2-tailed correlation. As different clones were measured at each time step, the analysis of possible correlations was restricted to clones from the end of the long-term experiment. Analysis was done separately for each species. The correlation was tested using clones from all treatments.

## Results

**Figure 1:**
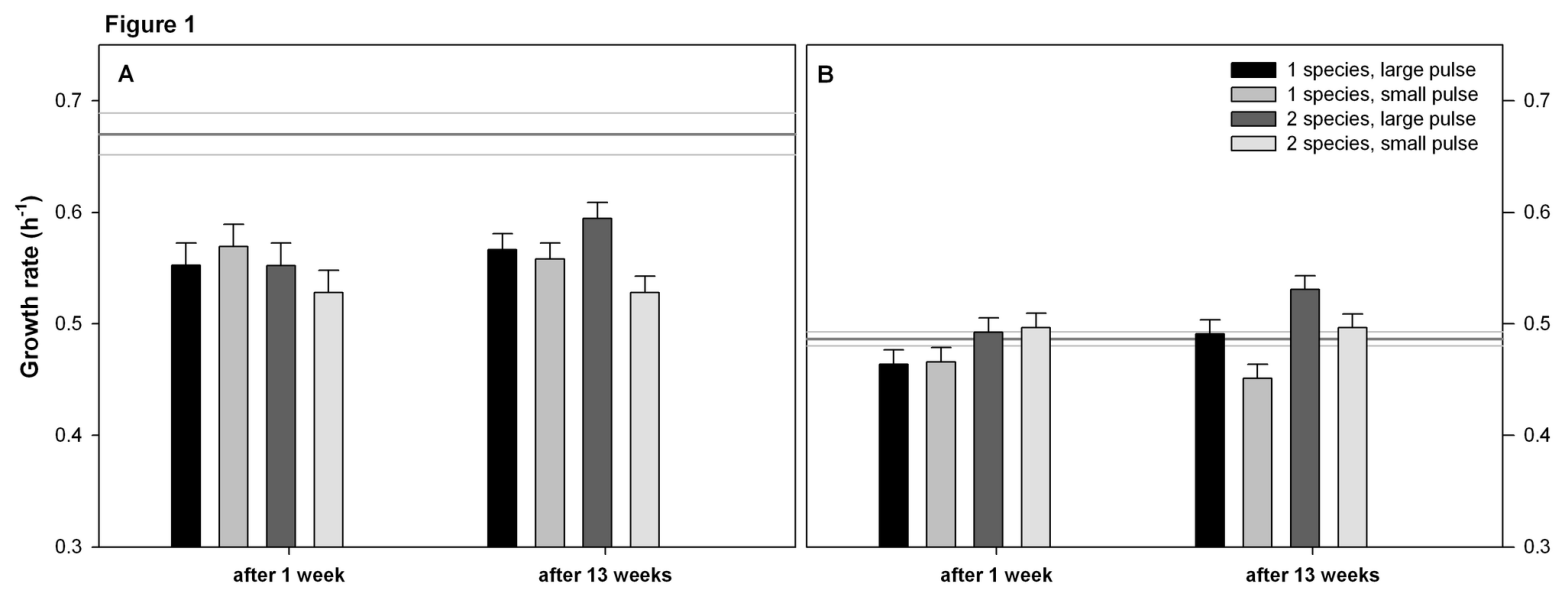
Maximum growth rate of A) *Serratia marcescens* B) *Novosphingobium capsulatum*. Growth rates were measured during a weeklong fitness assay. The bars show the estimated marginal mean growth rates (h^-1^) based on the GLMM + SE. The sampling time of bacterial clones is indicated on the x-axis: after 1 week = bacteria have a weeklong evolutionary history in the pulsed resource environment; after 13 weeks = evolutionary history is 13 weeks. The reference lines on the background indicate the mean maximum growth rate (dark grey line) ± SE (light grey lines) of the ancestral clones. Treatments: 1 species = species has grown in monocultures; 2 species = species has grown in two-species community; large pulse = 99,9 % of the total batch culture volume was renewed weekly; small pulse = 70% of the batch culture volume was renewed weekly.

**Figure 2:**
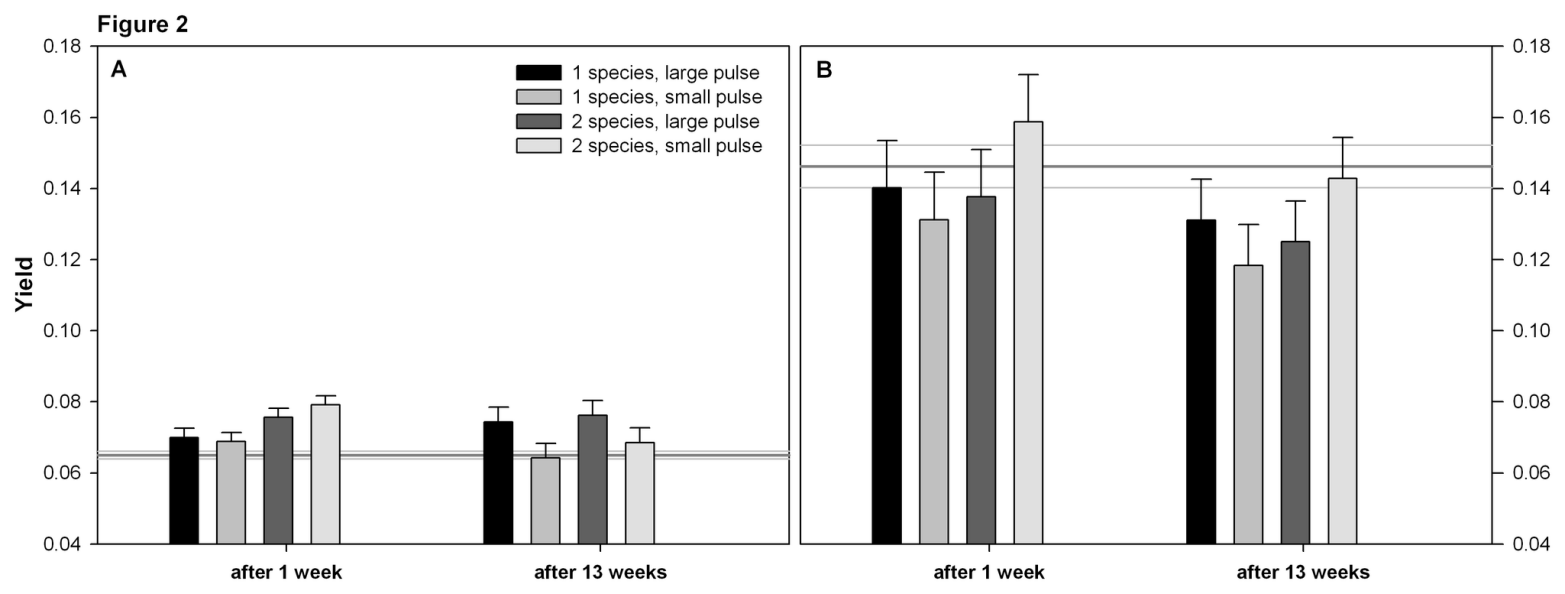
Yield of A) *Serratia marcescens* B) *Novosphingobium capsulatum*. Yield is measured as optical density, and corresponds to the total maximum biomass measured during a weeklong fitness assay. The bars show the estimated marginal mean yield based on the GLMM + SE in all treatments. Treatments are the same as in Figure 1.

**Figure 3:**
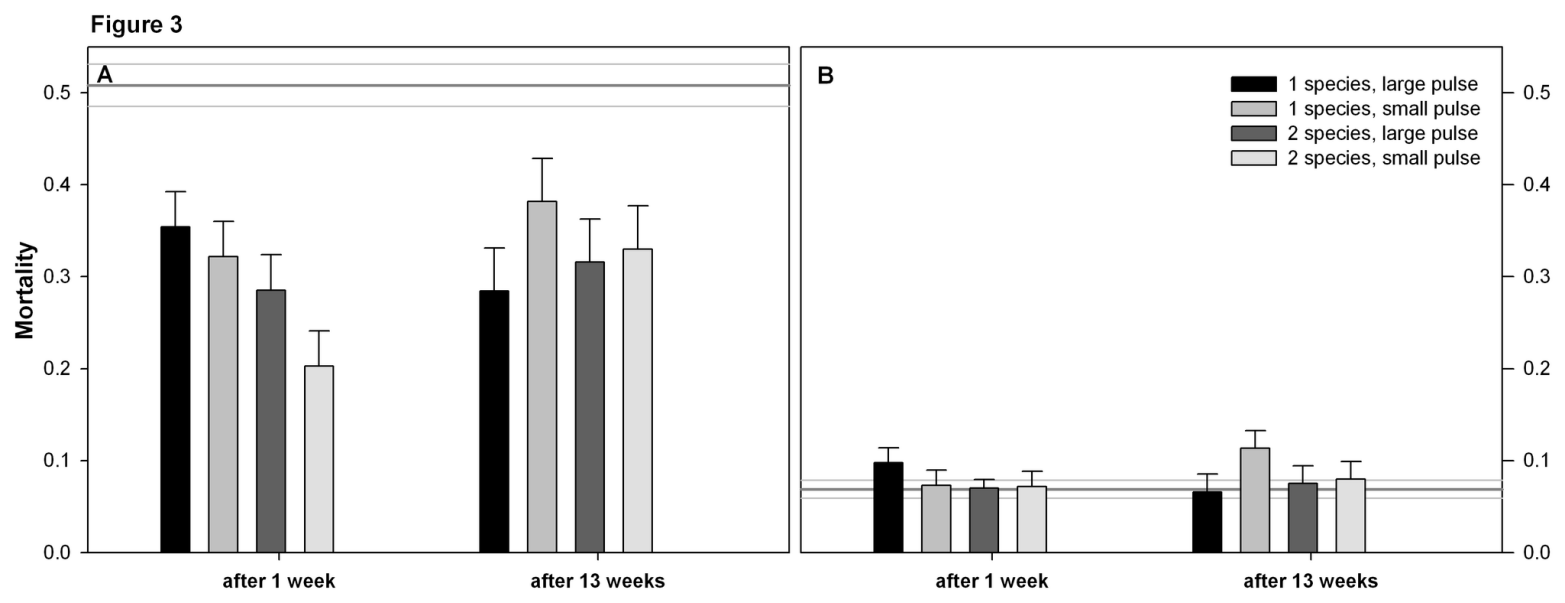
Mortality of A) *Serratia marcescens* B) *Novosphingobium capsulatum*. The mortality is calculated as proportional reduction in biomass during a weeklong fitness assay. The bars show the estimated marginal mean mortality based on the GLMM + SE in all treatments. Treatments are the same as in Figure 1.

**Figure 4:**
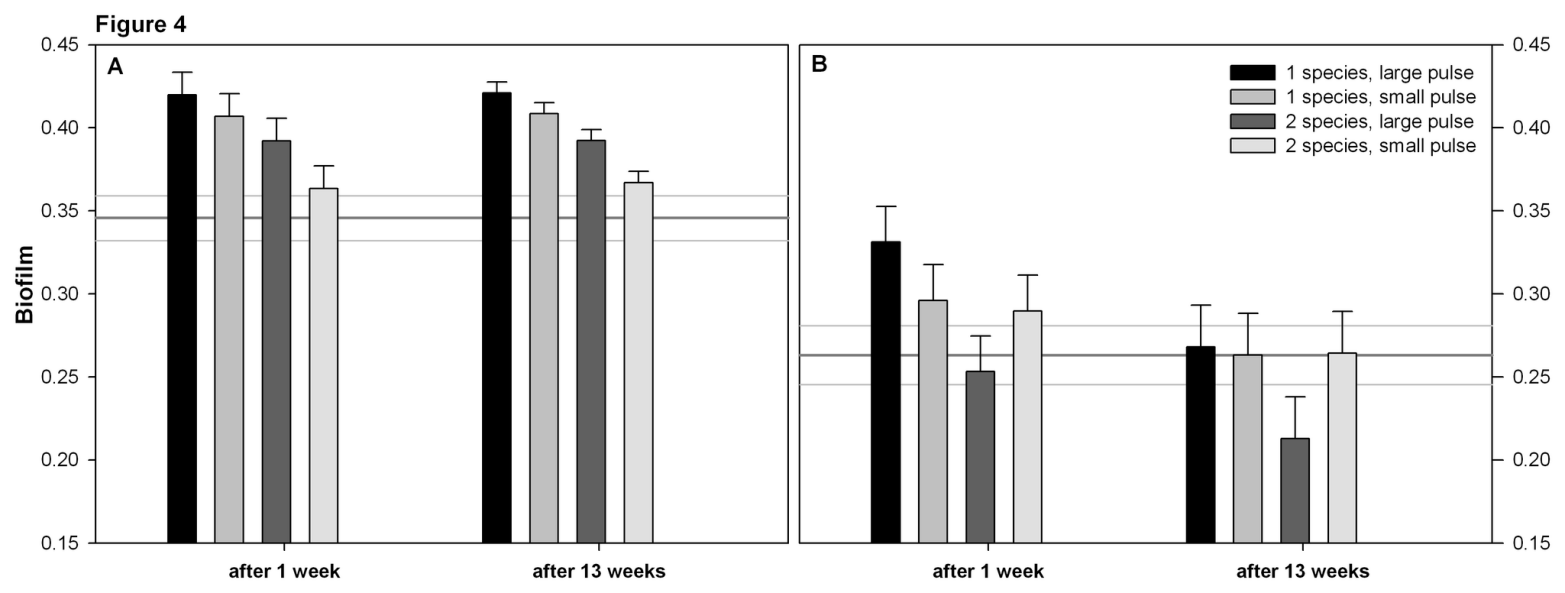
Biofilm production of A) *Serratia marcescens* B) *Novosphingobium capsulatum*. Biofilm amount was measured at the end of a weeklong fitness assay. The bars show the estimated marginal mean biofilm amount based on the GLMM + SE in all treatments. Treatments are the same as in Figure 1.

The ancestor clones of *N. capsulatum* had a lower maximum growth rate, higher yield and lower mortality than the ancestor *S. marcescens* (compare the reference lines between panels A and B in Figure 1 for growth rate, Figure 2 for yield, and Figure 3 for mortality). The ancestor *S. marcescens* produced more biofilm than *N. capsulatum* (Figure 4 A, B).

### Comparison of evolved strains to their common ancestor

#### Growth rate

Based on the fitness assays, the evolved *S. marcescens* clones from all but one treatment had a slower mean growth rate than their common ancestor (Table 1, Figure 1 A). Only the *S. marcescens* clones that had co-evolved with *N. capsulatum* for 13 weeks in large resource pulse environment did not differ significantly from their ancestor (Table 1). The mean growth rate of evolved *N. capsulatum* clones did not differ from their ancestor clones (Table 1, Figure 1 B).

**Table 1:** The pair-wise comparisons of evolved bacterial clones from different treatments and two sampling times to ancestor clones.

		***Serratia marcescens***						***Novosphingobium capsulatum***					
		**anc vs. 1 week^1^**			**anc vs. 13 weeks^2^**			**anc vs. 1 week**			**anc vs. 13 weeks**		
		**t**	**df**	**p**	**t**	**df**	**p**	**t**	**df**	**p**	**t**	**df**	**p**
**Growth^3^**	1 species^4^, large pulse^5^	**4.3**	**6.4**	**0.005**	**4.4**	**7.1**	**0.003**	1. 6	5.2	0.179	0.4	5.3	0.749
	1 species, small pulse^6^	**3.7**	**6.4**	**0.010**	**4.8**	**7.1**	**0.002**	1.4	5.2	0.213	2.5	5.3	0.052
	2 species^7^, large pulse	**4.3**	**6.4**	**0.005**	3.2	7.1	0.015	0.4	5.2	0.695	3.2	5.3	0.024
	2 species, small pulse	**5.2**	**6.4**	**0.002**	**6.0**	**7.1**	**0.001**	0.7	5.2	0.513	0.7	5.3	0.496
**Yield^8^**	1 species, large pulse	1.8	4.9	0.152	2.3	4.3	0.094	0.4	5.1	0.697	1.2	5.3	0.297
	1 species, small pulse	1.4	4.9	0.248	0.2	4.3	0.855	1.0	5.1	0.352	2.1	5.3	0.085
	2 species, large pulse	3.8	4.9	0.019	2.6	4.3	0.059	0.6	5.1	0.582	1.6	5.3	0.163
	2 species, small pulse	**5.0**	**4.9**	**0.007**	0.8	4.3	0.456	0.9	5.1	0.429	0.3	5.3	0.806
**Mortality^9^**	1 species, large pulse	3.4	5.6	0.018	**4.3**	**5.2**	**0.008**	1.5	5.5	0.189	0.1	5.2	0.902
	1 species, small pulse	**4.2**	**5.6**	**0.009**	2.4	5.2	0.060	0.2	5.5	0.824	2.1	5.2	0.095
	2 species, large pulse	**5.0**	**5.6**	**0.004**	3.7	5.2	0.014	0.1	5.5	0.928	0.3	5.2	0.783
	2 species, small pulse	**6.8**	**5.6**	**0.001**	3.4	5.2	0.019	0.2	5.5	0.876	0.5	5.2	0.636
**Biofilm^10^**	1 species, large pulse	**3.8**	**6.6**	**0.009**	**4.9**	**8.2**	**0.001**	2.4	6.2	0.050	0.2	5.9	0.880
	1 species, small pulse	3.1	6.6	0.021	**4.1**	**8.2**	**0.004**	1.2	6.2	0.281	0.003	5.9	0.998
	2 species, large pulse	2.4	6.6	0.056	3.0	8.2	0.017	0.4	6.2	0.734	1.6	5.9	0.164
	2 species, small pulse	0.9	6.6	0.403	1.4	8.2	0.206	1.0	6.2	0.377	0.04	5.9	0.972

The difference between ancestor and evolved strain is considered significant when p < 0.0125.

^1^ Comparison of evolved bacterial clones after one week to their ancestral clones.

^2^ Comparison of evolved bacterial clones after 13 weeks to their ancestral clones.

^3^ Maximum growth rate.

^4^ Bacterial clones have grown in monocultures.

^5^ Large resource pulse, where 99,9% of the total volume was renewed weekly.

^6^ Small resource pulse, where 70% of the total volume was renewed weekly.

^7^ Bacterial clones have grown in two-species communities.

^8^ Maximum biomass produced during one week.

^9^ Biomass reduction after population has reached its maximum biomass [(yield - end biomass)/yield].

^10^ Biofilm produced during a week.

#### Yield

*Serratia marcescens* clones that had co-evolved with *N. capsulatum* in small resource pulse environment for one week had higher yield than their ancestor (Table 1). The yield of the evolved *S. marcescens* clones from other treatments (Table 1, Figure 2 A) or the yield of the evolved *N. capsulatum* clones did not differ from their ancestor (Table 1, Figure 2 B).

#### Mortality

*Serratia marcescens* clones from most treatments had lower mortality than their ancestor (Table 1, Figure 3 A). The mortality of the evolved *N. capsulatum* clones did not differ from the mortality of the ancestor clones (Table 1, Figure 3 B).

#### Biofilm

*Serratia marcescens* clones from monocultures produced more biofilm than their ancestor when they had evolutionary history in large resource pulse environment for 1 or 13 weeks or small resource pulse environment for 13 weeks (Table 1, Figure 4 A). The biofilm production of *N. capsulatum* clones did not differ from their ancestor (Table 1, Figure 4 B).

### Evolutionary changes during the experiment

The mortality of *S. marcescens* decreased during the long-term experiment only if clones had grown in monocultures and experienced large resource pulses (time x pulse interaction in Table 2, Figure 3 A). *Serratia marcescens* clones from monocultures produced more biofilm than clones with evolutionary history in two-species communities (diversity effect in Table 2, Figure 4 A).

**Table 2:** Treatment effects on measured fitness traits based on GLMM.

***S. marcescens***	**F**	**df**	**p**		***N. capsulatum***	**F**	**df**	**p**	
**Time**					**Time**				
Growth	0.987	8	0.350		Growth	2.630	8	0.143	
Yield	1.218	8	0.302		Yield	3.436	8	0.100	
Biofilm	0.131	8	0.727		Biofilm	**17.708**	**8**	**0.003**	1w > 13w
Mortality	2.971	8	0.123		Mortality	1.304	8	0.287	
**Pulse**					**Pulse**				
Growth	2.344	8	0.164		Growth	2.945	8	0.124	
Yield	2.265	8	0.171		Yield	0.171	8	0.690	
Biofilm	4.207	8	0.074	large > small	Biofilm	0.318	8	0.588	
Mortality	0.001	8	0.982		Mortality	0.532	8	0.487	
**Diversity**					**Diversity**				
Growth	0.648	8	0.444		Growth	**13.328**	**8**	**0.006**	together > alone
Yield	4.645	8	0.063	together > alone	Yield	1.078	8	0.329	
Biofilm	**13.276**	**8**	**0.007**	alone > together	Biofilm	2.644	8	0.143	
Mortality	1.973	8	0.123		Mortality	0.085	8	0.778	
**Pulse × Diversity**					**Pulse × Diversity**				
Growth	3.402	8	0.102		Growth	0.041	8	0.844	
Yield	0.476	8	0.510		Yield	2.112	8	0.184	
Biofilm	0.542	8	0.483		Biofilm	2.250	8	0.172	
Mortality	0.813	8	0.394		Mortality	1.949	8	0.200	
**Time × Diversity**					**Time × Diversity**				
Growth	0.753	8	0.411		Growth	0.663	8	0.439	
Yield	1.127	8	0.319		Yield	0.058	8	0.815	
Biofilm	0.003	8	0.957		Biofilm	0.626	8	0.452	
Mortality	3.819	8	0.086		Mortality	4.116	8	0.077	
**Time × Pulse**					**Time × Pulse**				
Growth	2.190	8	0.177		Growth	**6.499**	**8**	**0.034**	
Yield	4.831	8	0.059		Yield	0.067	8	0.802	
Biofilm	0.040	8	0.846		Biofilm	1.377	8	0.274	
Mortality	**6.952**	**8**	**0.030**		Mortality	3.004	8	0.121	

Treatments: time = how long time species have been in the resource pulse environment (1w = for 1 week, 13w = for 13 weeks); pulse = the magnitude of the weekly resource pulse (large = 99.9% of the total volume was renewed with fresh resources, small = 70% of the total volume was renewed); diversity = indicates whether bacterial species grew in a monoculture (alone) or in a two-species community (together) during the long-term experiment. Fitness traits are the same as in Table 1. Significant differences with p < 0.05 are highlighted.

The growth rate of *N. capsulatum* increased in large resource pulse treatment (time x pulse interaction in Table 2, Figure 1 B), and clones with evolutionary history in two-species communities grew faster than clones evolved in monocultures (diversity effect in Table 2, Figure 1 B). Also clonal variation in growth rate of *N. capsulatum* increased during the experiment (0.033 ± 0.003 vs. 0.066 ± 0.012, EMM of SD in growth rate after 1 week vs. 13 weeks ± SE, respectively; F_8.0_ = 8.823, p = 0.018). During the long term experiment the biofilm production of *N. capsulatum* clones decreased (time effect in Table 2, Figure 4 B).

### Correlations between traits in evolved clones

The growth rate *S. marcescens* clones with 13 weeks evolutionary history in pulsed resource environment was positively correlated with mortality (r = 0.399, N = 162, p < 0.001), but there was no correlation between growth rate and yield (r = -0.075, N = 162, p = 0.342). There was no correlation between the growth rate of evolved *N. capsulatum* clones from week 13 and mortality (r = -0.002, N = 164, p = 0.979), or growth rate and yield (r = -0.101, N = 164, p = 0.197).

## Discussion

We studied the effects of low frequency i.e. inter-generational resource pulses and community structure on the evolution of fitness related traits and trait variation using two-species bacterial model system. The trait measurements were done at clonal level to reveal changes in genetic variation. The evolutionary response to competition and the resource pulse magnitude were different depending on the species: the growth rate of bacterium *Serratia marcescens* decreased and survival increased in most treatments, but similar, general changes were not found in any measured trait of *Novosphingobium capsulatum*. Both the competitive environment, and the magnitude of the resource pulses caused evolutionary changes in *N. capsulatum*. Clonal variation increased only in *N. capsulatum* growth rate. In other measured traits of either species the clonal variation did not change.

The general finding of increased survival in *S. marcescens* clones is in line with other experiments suggesting that the mechanisms enabling survival of bacteria during low resource conditions can evolve in periodically fluctuating environments [40,41]. In low resource conditions evolutionary changes can enable bacteria to utilize new resource compounds including metabolites produced by other bacteria (cross-feeding interaction [40,42–45]), or even the dead biomass of their own species [41]. One potential scenario in co-cultures of *S. marcescens* and *N. capsulatum* was that species would evolve a mutualistic or ammensalistic cross-feeding interaction. In that case, when bacterial clones adapted to presence of another species were grown in monocultures, the growth rates or survival could be low. According to our earlier study, a cross-feeding interaction is possible between *S. marcescens* and *N. capsulatum*. When the growth rate of each species was tested in a sterile-filtered medium where the other species had consumed the resources for 1 to 168 h, the growth rates in consumed medium were occasionally faster than in unconsumed medium [27]. *Serratia marcescens* had a relatively high growth rate in medium consumed by *N. capsulatum* for 1 to 10 h, whereas *N. capsulatum* had high growth rates in medium consumed by *S. marcescens* for 30 to 50 h [27]. However, results presented here show that when these species grew together for prolonged time, they did not evolve to be dependent on a cross-feeding interaction. In fitness assays where bacterial clones were grown in monocultures, *S. marcescens* clones had a better survival and slower growth rate than their ancestor irrespective of their evolutionary history. Thus, the absence of other species did not result in increased mortality nor does it explain the decreased growth rate of *S. marcescens*. Furthermore, we found changes in only two traits in *N. capsulatum* clones: growth rate and biofilm production. In fitness assays the *N. capsulatum* clones that had evolved together with *S. marcescens* grew faster than clones evolved in monocultures. The relaxation of interspecific interaction actually enabled evolved *N. capsulatum* clones to grow faster, which supports the view that the interaction between species is competitive, not beneficial. This is in accordance with our earlier finding that both species produce larger population sizes in monocultures than when grown together [28].

Interspecific competition had different effects on the studied species. The evolved clones from two species communities had higher growth rate (*N. capsulatum*) or produced less biofilm (*S. marcescens*) than the clones of the same species from monocultures. The importance of competitive interactions in environments where resource availability fluctuates has been under debate for decades (examples of competition and resource fluctuations discussion in plant communities [11,46–49]). Also, the effect of resource fluctuations on community composition and competitive interactions has received both theoretical [5,9,18,50,51] and experimental attention [26,28,52–54]. The intensity of competition and the competitive ranking of species in a community may vary depending on resource availability [51]. Here resource availability varied depending on the resource pulse magnitude and the resource consumption rate during the weeklong interpulse period. Different traits are beneficial during the resource pulses and longer interpulse periods [9,11]. In our system it is plausible that the quick growth to high population size during resource abundance, right after the pulse, gives a competitive edge for *S. marcescens* over *N. capsulatum*. Furthermore, competition between bacterial species may include interference where bacteria secrete toxic substances that hinder the growth of other cells in close proximity [55]. The relaxation of interspecific competition in fitness assays would explain the high growth rates of evolved *N. capsulatum* clones from two-species communities.

The increase in *S. marcescens* survival in pulsed resource environments indicates adaptation to the low resource conditions during the interpulse period. When organisms have to adapt to changing environmental conditions, competitive interactions can in theory either speed up or slow down the rate of evolution depending on the direction of selection pressures caused by competition and environmental change [31]. Competition may have affected the evolutionary change in *S. marcescens* survival in two distinct ways. First, as the survival of *S. marcescens* clones did not improve when they had co-evolved with another species, it is possible that the simultaneous allocation of resources to coping with interspecific competition and to adaptation to pulsed resource environment hindered the evolutionary increase in survival [31]. Alternatively, intraspecific competition and large resource pulses may have caused parallel selection pressures. This scenario would explain why survival increased in monocultures [31]. These two explanations are not mutually exclusive.

In our experimental setting, the selection for survival at low resource conditions was strong as the individuals that died during the interpulse could obviously not grow and exploit the next resource pulse. Goldberg and Novoplansky [11] have formulated a two-phase resource hypothesis to describe plant interactions along productivity gradients. According to this hypothesis, the survival during interpulse is a crucial biological challenge. The survival of *S. marcescens* increased substantially already after the first week, and thereafter continued to increase only in monocultures that experienced large resource pulses. The increased survival of *S. marcescens* in environments with low frequency resource pulses is in concordance with predictions of the two-phase resource hypothesis. In addition to the increased survival, also the ability to produce high yield can indicate tolerance to low resource conditions. High yield can be achieved by several mechanisms in an environment with rare resource pulses. An ability to utilize resources also during the interpulse, an efficient growth right after the pulse, and an ability to survive for prolonged time periods, or the combination of these can all result in high yield. The evolved clones did not produce more yield than their ancestor; neither did the yield production differ between treatments. We measured yield and survival based on turbidity, which makes the separation of living and dead cells impossible. Thus, also the dead biomass potentially affects the yield estimate. However, we have measured these same strains in a separate experiment where population dynamics were based on colony counts in weeklong fitness assays. These assays showed that the evolved strains of both *S. marcescens* and *N. capsulatum* had better survival and higher end population sizes than their ancestors [28]. Both data from the population level measurements and clonal measurements here support the interpretation that better survival during the interpulse is an evolutionary response to environmental conditions where resources become available rarely.

The increased survival of evolved *S. marcescens* clones in comparison to their ancestor affected also growth as the evolved clones had a slower growth rate than the ancestor. Among the *S. marcescens* clones with 13 weeks history in pulsed resource environment increase in growth rate was correlated with increase in mortality. This finding indicates a potential trade-off between growth rate and survival in *S. marcescens*. Previous population level measurements on the same study system did not reveal trade-offs between life-history traits [28]. Trade-offs are thought to be inevitable as no organism can perform well in everything and everywhere (reviewed by [56,57], but see [58]). It has been suggested that especially yield (K) and growth rate (r) show this kind of trade-off [14,15]. However, we did not detect any sign of a general trade-off between yield and growth rate. On the contrary, the growth rate of *N. capsulatum* increased only in the large resource pulse treatment if it had grown with *S. marcescens*, and *N. capsulatum* yield or survival did not change during the experiment. This is probably due to difference in species growth strategies: the ancestral strain of *S. marcescens* grows more rapidly during resource abundance, but has a lower survival and yield during resource scarcity than *N. capsulatum*. The ancestor strain of *N. capsulatum* survives in low resource environments, and further improvement may not be under strong selection. Very likely these species experienced different selection pressures during the long-term experiment, and thus also the evolutionary changes in measured life-history traits were species-specific.

We did detect evolutionary changes in the trait means but clonal variation changed only in one trait: the clonal variation in *N. capsulatum* growth rate increased during the experiment. This contradicts the common finding of adaptive radiation in bacterial populations grown in microcosms [25]. The change in mean values without combined change in variation could indicate directional selection. Furthermore, the clonal variation in measured traits within each treatment was in general low (data not shown), which decreases the possibility of clonal variation blurring the detection of possible trade-offs between measured traits. Low variation at clonal level also suggests, that selection and/or genetic, metabolic or other physiological constraint limited the evolutionary response of *S. marcescens* in all traits and *N. capsulatum* in all other traits except growth rate. This gives further support to our interpretation that *S. marcescens* trades-off survival during resource scarcity with fast growth rate during resource abundance, whereas *N. capsulatum* does not (or it is not manifested in these environmental conditions).

As an additional fitness estimate we measured how much biofilm bacteria formed during a week. A general trend for both species was an initial increase in biofilm production: most clones with a weeklong history in experimental environment produced more biofilm than their ancestor. Thereafter the biofilm production of *S. marcescens* did not change: after 13 weeks all evolved *S. marcescens* clones produced on average more biofilm than the ancestor. During the long-term experiment the *N. capsulatum* biofilm production decreased in all treatments, but still the evolved clones produced more biofilm than their ancestors. This is interesting as we expected a decrease in biofilm production. The selection should have been against biofilm forming: In the weekly resource renewals the population was always transferred from the liquid phase to a new microcosm leaving most of the biofilm to the previous microcosm (see Methods). However, some biofilm forming cells may have been included in the transfer. The microcosms were shaken before resource renewal and thus parts of the biofilm may have detached from the vessel’s walls and got mixed in the liquid. Furthermore, it is known that the detachment of biofilms is also a bacterial dispersal strategy [59,60]. Alternatively, it could be speculated that the qualitative changes in the growth environment due to bacterial metabolism and resource consumption may also influence biofilm production [59–62]. We know that during one week the quality of the resource environment changes and affects bacterial growth rates [27]. However, the effect of changing resource conditions on biofilm production is not a likely explanation here, as there was no consistent difference in biofilm formation between clones from small and large resource pulse environments.

Though the survival of biofilm forming cells in populations during the long-term experiment could be attributed to the mixing of microcosms, we found also changes in biofilm production that are most likely adaptive. The *S. marcescens* clones evolved in monocultures produced more biofilm than those from two-species communities. Growth as biofilm can be a way of escaping competition between free-swimming cells and could thus promote diversity within a population and enable coexistence in a community [61]. It has been shown that for example commensalism can evolve in two-species bacterial communities of *Pseudomonas putida* and *Acinetobacter* sp. biofilms but not if the same species grow together as free swimming cells [63]. *Serratia marcescens* biofilm growth is a commonly found adaptation against stressors, such as antibiotics and predators [37,64]. Here the *S. marcescens* biofilm production was possibly a response to the intensity of competition suggesting that for *S. marcescens* intraspecific competition was more intense than interspecific competition.

To summarize, we have exemplified the importance of interactions within and between species in shaping the evolution of fitness related traits in bacterial populations exposed to intergenerational low frequency resource pulses. The quantitative and qualitative differences in the two resource pulse regimes caused different selection pressures on bacterial traits. The evolutionary responses of bacterial clones to periodic resource fluctuations were species specific, and depended on both the abiotic and the biotic environment. The increased survival and decreased growth rate in clones of the copiotroph species (*S. marcescens*) were most likely a response to resource fluctuations. In contrast, the clones of oligotroph species (*N. capsulatum*) increased growth rate but only if they had experienced interspecific competition and large resource pulses. Our interpretation is that interspecific competition caused increase in *N. capsulatum* growth rates, whereas intraspecific competition or abiotic environmental factors caused evolutionary changes in *S. marcescens* survival and biofilm production. This suggests that the life history differences of the interacting species, in our case copiotroph vs. oligotroph strategies, are important for predicting how species evolve in fluctuating environments. Though it has been suggested that facilitative or mutualistic interactions in e.g. nutrient cycling enable coexistence in microbial communities [65], the ability to coexist does not rule out competition between species [66]. Recently it has been shown experimentally that competitive interactions potentially dominate microbial interactions [29,30]. Our study is among the first showing that evolution in pulsed resource environment is strongly modified by biotic interactions, especially by the presence of competitor species.
